# Polymorphism of viral dsRNA in *Xanthophyllomyces dendrorhous *strains isolated from different geographic areas

**DOI:** 10.1186/1743-422X-6-160

**Published:** 2009-10-08

**Authors:** Marcelo Baeza, Mario Sanhueza, Oriana Flores, Vicente Oviedo, Diego Libkind, Víctor Cifuentes

**Affiliations:** 1 Laboratorio de Genética, Departamento de Ciencias Ecológicas, Facultad de Ciencias, Universidad de Chile, Santiago, Chile; 2 Laboratorio de Microbiología Aplicada y Biotecnología. Bariloche, Río Negro, Argentina

## Abstract

**Background:**

Strains of the astaxanthin producing yeast *Xanthophyllomyces dendrorhous *have been isolated from different cold regions around the earth, and the presence of double stranded RNA (dsRNA) elements was described in some isolates. This kind of viruses is widely distributed among yeasts and filamentous fungi and, although generally are cryptic in function, their studies have been a key factor in the knowledge of important fungi. In this work, the characterization and genetic relationships among dsRNA elements were determined in strains representatives of almost all regions of the earth where *X. dendrorhous *have been isolated.

**Results:**

Almost all strains of *X. dendrorhous *analyzed carry one, two or four dsRNA elements, of molecular sizes in the range from 0.8 to 5.0 kb. Different dsRNA-patterns were observed in strains with different geographic origin, being L1 (5.0 kb) the common dsRNA element. By hybridization assays a high genomic polymorphism was observed among L1 dsRNAs of different *X. dendrorhous *strains. Contrary, hybridization was observed between L1 and L2 dsRNAs of strains from same or different regions, while the dsRNA elements of minor sizes (M, S1, and S2) present in several strains did not show hybridization with neither L1 or L2 dsRNAs. Along the growth curve of UCD 67-385 (harboring four dsRNAs) an increase of L2 relative to L1 dsRNA was observed, whiles the S1/L1 ratio remains constant, as well as the M/L1 ratio of Patagonian strain. Strains cured of S2 dsRNA were obtained by treatment with anisomycin, and comparison of its dsRNA contents with uncured strain, revealed an increase of L1 dsRNA while the L2 and S1 dsRNA remain unaltered.

**Conclusion:**

The dsRNA elements of *X. dendrorhous *are highly variable in size and sequence, and the dsRNA pattern is specific to the geographic region of isolation. Each L1 and L2 dsRNA are viral elements able to self replicate and to coexist into a cell, and L1 and S2 dsRNAs elements could be part of a helper/satellite virus system in *X. dendrorhous*.

## Background

A wide range of microorganisms have extrachromosomal genetic elements (EGEs) as circular or linear double stranded DNA (dsDNA) or double stranded RNA molecules (dsRNA) encapsidated in cytoplasmically inherited virus like particles (VLPs) [1,2]. In only few cases these dsRNAs have been associated with a detectable phenotype in the host, such as virulence [3,4]. The yeast dsRNA viruses most studied are those responsible for production of mycocin (killer system) from strains of *Ustilago maydis *and *Saccharomyces cerevisiae *[5-10]. Most strains of *S. cerevisiae *carry L-A dsRNA (4.6 kb, belonging to the *Totiviridae *virus family) encoding structural proteins (Gag and GagPol) that are necessary for its replication and packaging. In addition, strains with killer phenotype (K^+^) carry an additional M dsRNA (1.6 to 1.8 kb), which codes for a mycocin and self-immunity. M dsRNA is called a "satellite virus" because it utilizes the proteins encoded by L-A ("helper virus") for its packaging and replication. In other *Saccharomyces *yeasts, the M dsRNA showed a high variation in size and according to phenotypic analysis are cryptic [11,12]. In strains of the astaxanthin-producing yeast *Xanthophyllomyces dendrorhous*, the presence of EGEs of dsRNA, dsDNA, or both has been reported [13]. Isolates of this yeast were obtained from tree exudates in cold climate areas of Japan, Russia, Alaska, and Finland [14,15] and recently, new strains have been isolated from areas of Germany [16] and Argentina [17,18]. Analysis of strains isolated from Finland and Japan showed that they carry zero and four dsRNAs, respectively, of lengths between 0.8 and 4.3 kb [15,19-21], but there are no data about the dsRNA content of *X. dendrorhous *strains from other areas. The sequences and functions of these viruses are still unknown, and only a slightly effect on host reproduction and fitness has been reported [19]. In this paper, strains of *X. dendrorhous *isolated from Japan, Russia, Finland, Alaska, and South America were analyzed in relation to their content of dsRNA elements, and the genomic similarity among those elements was evaluated by hybridization. Furthermore, the relative abundance of dsRNAs was determined in wildtype and cured strains.

## Results

### dsRNA polymorphisms in *X. dendrorhous *strains isolated from different geographic areas

**Figure 1:**
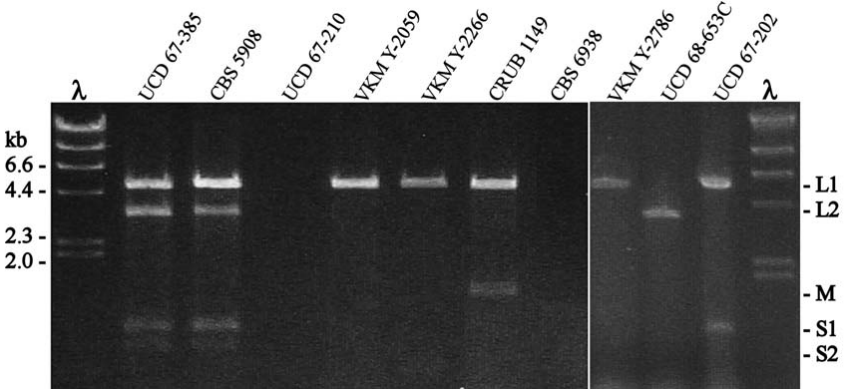
**dsRNA pattern in *X. dendrorhous *strains**. The total RNA was isolated from cultures of each strain, treated with nuclease S1 and separated in 1% agarose gel electrophoresis. λ, λ *Hind*III DNA marker.

The total RNA was extracted from late exponential phase cultures of nine strains of *X. dendrorhous *isolated from different geographic areas, and one strain of *Phaffia rhodozyma *(the imperfect phase of *X. dendrorhous*) (Table 1). To establish the presence of dsRNA elements, the samples of RNA obtained were treated with several enzymes and later subjected to electrophoresis. In this way, the dsRNA elements were identified by their resistance to treatments with DNase I, RNase H and Nuclease S1, and to RNase A at high ionic strength; and by their sensibility to treatment with RNase A at low ionic strength. Results obtained after treatment with nuclease S1 are shown in Fig 1. Almost all strains analyzed carried at least one dsRNA element, and a high variability in the dsRNA pattern of different *X. dendrorhous *strains was observed (Fig 1). The *P. rhodozyma *UCD 67-210 and *X. dendrorhous *CBS 6938 strains have no dsRNAs. The other *X. dendrorhous *strains have one (VKM Y-2786, VKM Y-2059, VKM Y-2266, and UCD 68-653C), two (UCD 67-202, CRUB 1151, and CRUB 1149), or four (UCD 67-385 and CBS 5908) dsRNA elements. Based on the molecular size and hybridization results (see below) five different types of dsRNAs were found and were renamed as L1 (5.0 kb), L2 (3.7 kb), M (1.4 kb), S1 (0.9 kb), and S2 (0.8 kb), for **L**arge, **M**edium and **S**mall (Table 2). The L1 is the common dsRNA element among the dsRNA-harboring strains, with the exception of UCD 68-653C, which only carries the L2 dsRNA. The dsRNAs of minor size (S1 and S2) were observed only in *X. dendrorhous *strains isolated from Japan (UCD 67-385, CBS5908, and UCD 67-202). The M dsRNA was found only in CRUB strains (Argentina's Patagonia) and has molecular size (1.4 kb) not previously described in *X. dendrorhous*.

**Table 1:** Yeast strains used in this work.

**Species**	**Strains**	**Other culture collections**	**Source**
*P. rhodozyma*	UCD 67-210	CBS 5905, ATCC 24202, VKM Y-2274	Exudate of *Fagus crenata*, Uehimi-yama, Kyoto, Japan.
*X. dendrorhous*	CBS 6938	VKM Y-2793, UCD 77-61, ATCC 96594	Sap of *Betula *sp. stumps, Finland.
	VKM Y-2059	ATCC 96814	Flux of *Betula verrucosa*, Moscow Region, Russia.
	VKM Y-2786	CBS 7918	Exudate of *Betula verrucosa*, Moscow Region, Russia.
	VKM Y-2266	UCD 76-18	Flux of *Betula verrucosa *Ehrh. Moscow Region, Russia.
	UCD 68-653C	ATCC 24228	Exudate of *Betula papyrifera*, Rainbow Lake, Kenai Peninsula, Alaska.
	CRUB 1149	-	Water from Lake Ilon, Patagonia, Argentina.
	CRUB 1151	-	Water from Lake Ilon, Patagonia, Argentina.
	UCD 67-385	ATCC 24230, CBS 7919 VKM Y-2791	Exudate of *Betula tauschii*, Shinkai, Kiso, Japan.
	CBS 5908	UCD 67-383, ATCC 24203, VKM Y-2790	Exudate of *Alnus japonica*, Shinkai, Kiso, Japan.
	UCD 67-202	ATCC 24229	*Cornus brachypoda*, Hiroshima, Japan.

The following abbreviations are used for microorganism culture collections: CBS, Centraalbureau voor Schimmelcultures, Utrecht, Netherlands; ATCC, American Type Culture Collection, Manassas, USA; UCD, Phaff Yeast Culture Collection, Department of Food Science and Technology, University of California at Davis, Davis, USA; VKM, The All-Russian Collection of Microorganisms, Moscow, Russia; CRUB, Centro Regional Universitario Bariloche, Bariloche, Argentina.

**Table 2:** dsRNA elements of *X. dendrorhous *strains.

**Strains**	**dsRNAs**			**Abbrev.^a^**
		
	**N°**	**Name**	**kb**	
UCD 67-210 ^b^	0	-	-	
CBS 6938	0	-	-	
VKM Y-2059	1	L1	5.0	2059L1
VKM Y-2786	1	L1	5.0	2786L1
VKM Y-2266	1	L1	5.0	2266L1
UCD 68-653C	1	L1	3.7	653L2

UCD 67-202	2	L1	5.0	202L1
		S1	0.9	
CRUB 1151	2	L1	5.0	
		M	1.4	
CRUB 1149	2	L1	5.0	1149L1
		M	1.4	1149M

UCD 67-385	4	L1	5.0	385L1
		L2	3.7	385L2
		S1	0.9	385S1
		S2	0.8	385S2
CBS 5908	4	L1	5.0	5908L1
		L2	3.7	5908L2
		S1	0.9	
		S2	0.8	

^a^, abbreviations employed in Fig 2. ^b^, type strain of *P. rhodozyma*.

### Dot-blot hybridizations and characterization of dsRNAs

**Figure 2:**
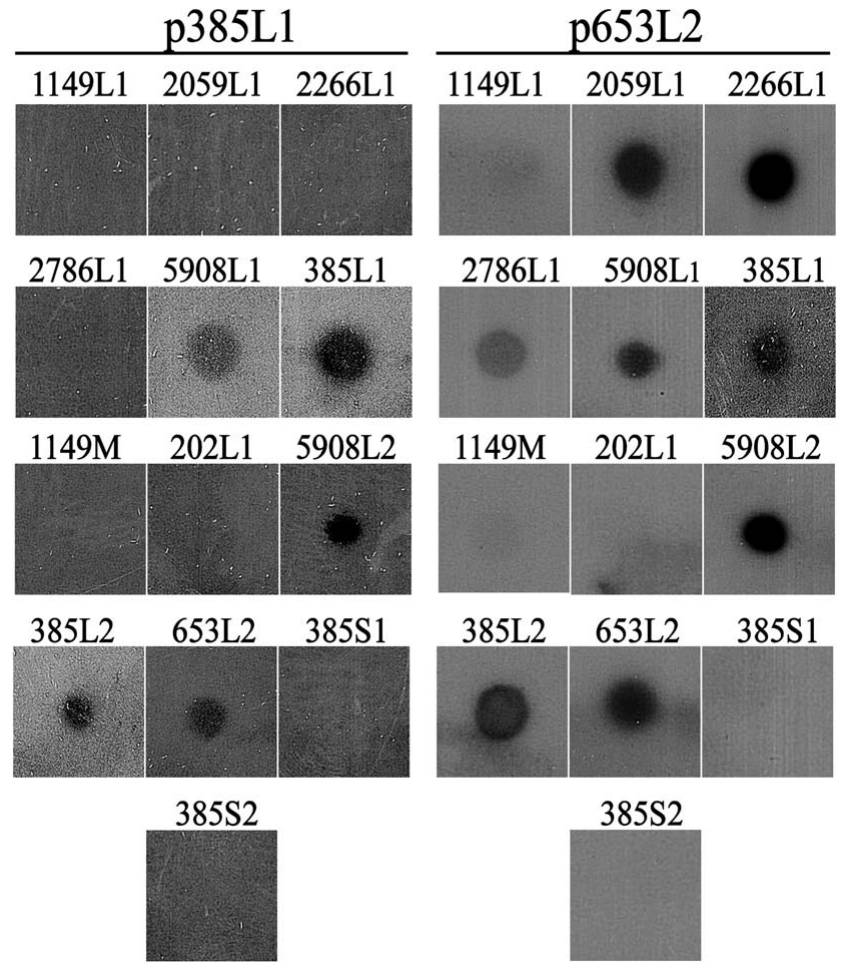
**Dot-blot hybridization**. Heat denatured dsRNAs were fixed onto nylon membranes and hybridized with radioactive cDNA probes synthesized from L1dsRNA of UCD 67-385 (p385L1) and L2 dsRNA from UCD 68-653C (p653L2). Assays were performed twice obtaining the same results. See Table 2 for abbreviations.

The similarity at genomic level among different dsRNA elements of *X. dendrorhous *strains was evaluated by cDNA-RNA hybridization. Radioactive probes were synthesized by reverse transcription with random hexamers using as template the L1 and L2 dsRNA elements of strain UCD 67-385 (probes p385L1 and p385L2, respectively), the L2 of UCD 68-653C (probe p653L2) and the L1 of VKM Y-2786 (probe p2786L1). These probes were used in hybridization experiments targeting heat denatured dsRNA elements purified from different strains of *X. dendrorhous *(Fig. 2 and Table 3). In hybridization assays with probe p385L1, positive signals were obtained with L1 dsRNA of CBS 5908 (5908L1), and with L2 dsRNA of strains UCD 67-385 (385L2), CBS 5908 (5808L2) and UCD 68-653C (653L2). The UCD 67-385 and CBS 5908 correspond to *X. dendrorhous *strains isolated from the same Japanese region but from different source, and the strain UCD 68-653C was isolated from Alaska (Table 1). The probe p653L2 hybridized with 385L1, 385L2, 5908L1 and 5908L2 dsRNAs, confirming the results obtained with the probe p385L1; and furthermore hybridized with L1 dsRNAs of Russian strains, with intensities as strong as the control (653L2) for 2059L2 and 2266L1, and a weaker signal for 2786L1. The dsRNA elements S1, S2, and those of UCD 67-202 and CRUB 1149 strains did not hybridized with any of the probes assayed (Fig 2, Table 3). Concordant results were obtained in hybridization assays with the probes p385L2 and p2059L1 (Table 3).

**Table 3:** Hybridization among dsRNA elements of *X. dendrorhous *strains isolated from different geographic areas.

		**Probe**			
		
**dsRNA element**	**Country - region**	**p385L1 (Japan-Kiso)**	**p653L2 (Alaska)**	**p385L2 (Japan-Kiso)**	**p2059L1 (Russia)**
L1	Japan - Kiso	+	+	+	-
	Japan - Hiroshima	-	+	-	nd
	Russia	-	+	-	+
L2	Japan - Kiso	+	+	+	-
	Alaska	+	+	+	+
M	Argentina	-	-	-	nd
S1 and S2	Japan	-	-	-	nd

nd, not determined. Radioactive probes were synthesized by reverse transcription with random primers using as template purified dsRNA elements from strains UCD 67-385 (p385L1 and p385L2), UCD 68-653C (p653C) and VKM Y-2059 (p2059L1).

### dsRNA elements along growth curve of *X. dendrorhous *and curing assays

**Figure 3:**
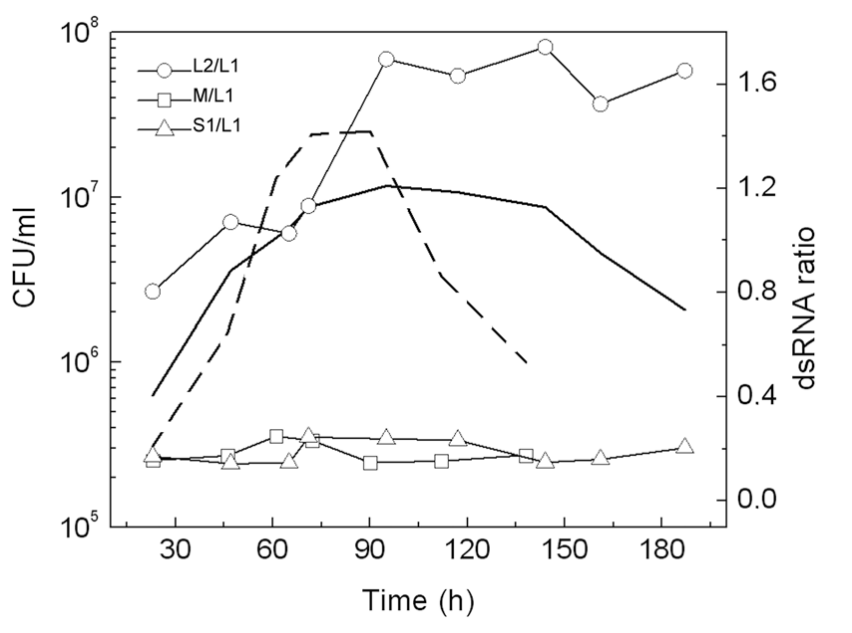
**Relative abundance of dsRNA elements in *X. dendrorhous *along the growth curve**. Growth curves of strains UCD 67-385 (continuous line) and CRUB 1149 (dashed line) were performed in YM medium at 22°C, and total RNA was extracted from culture samples collected at each point. The ratios of dsRNA elements L2/L1 and S1/L1 of UCD 67-385, and M/L1 of CRUB 1149, were calculated from the bands intensities of each dsRNA elements separated in agarose gels. The experiments were performed at least tree times and a representative curve for each strains is shown.

**Figure 4:**
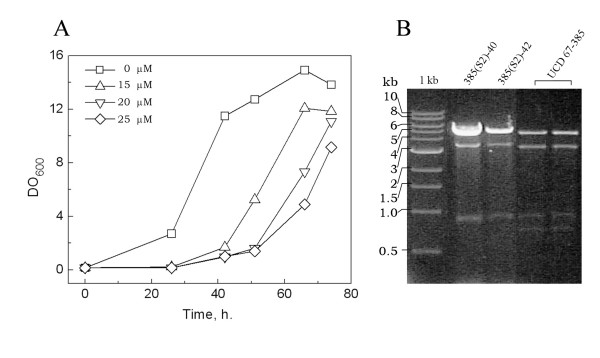
**Loss of S2 dsRNA by treatment with anisomycin**. Cultures of strain UCD 67-385 were made in YM broth supplemented with different concentrations of anisomycin (A). Culture samples were removed at 42 and 50 h and streaked for single colonies onto YM medium. The total RNA was extracted from cultures of each colony and the content of dsRNA elements was determined (B). The dsRNA pattern of two strains cured of S2 dsRNA [385(S2)-40 and 385(S2)-42] and uncured (UCD 67-385) strains are shown.

The total RNA were isolated from samples harvested at different phases of the growth curve of *X. dendrorhous *UCD 67-385 and CRUB 1149, and the relative abundance of dsRNA elements was determined (Fig. 3). In culture of the strain UCD 67-385, the amount of L2 relative to L1dsRNA (L2/L1 ratio) increased from 0.8 (middle-log phase) to 1.7 (stationary phase), while the S1/L1 ratio remains around 0.2 along the entire growth curve; as well as the S2/L1 ratio (not shown). The ratio of the two dsRNA elements of strain CRUB 1149 (M/L1ratio) show no variation along the growth curve, with ratio values around 0.2. These behaviors are independent of metabolic state of the yeast, because the same results were obtained in cultures performed in rich and minimal media supplemented with glucose or succinate that are fermentable and not fermentable carbon sources respectively (not shown). The ratios observed between L1 and S1 or S2 dsRNAs are similar to the behavior observed for the satellite and helper viruses of the killer system in *S. cerevisiae*, and furthermore in this yeast the elimination of the M dsRNA (satellite virus) results in an increase of the copy number of L dsRNA (helper virus) [22]. The viral dsRNAs of *S. cerevisiae *can be easily cured by treatment with sublethal doses of peptidyl-transferase inhibitors as anisomycin [23]. This drug was used to induce the loss of dsRNA elements in the strain UCD 67-385 of *X. dendrorhous*. Treatments of cultures with concentrations of anisomycin until 7.6 μM did not have any effect on the growth of *X. dendrorhous *(not shown), and only concentrations over 15 μM induce a growth delay (Fig 4A). Colonies were isolated from samples of cultures treated for 42 and 50 h with 15, 25 and 30 μM of anisomycin, inoculated in 10 ml of YM medium and incubated for 3 days at 22°C. Following, the content of dsRNA elements was determined for culture of each colony and, after the analysis of 400 colonies, only two clones that have been lost the S2 dsRNA were identified (Fig 4B). In these S2-cured clones a marked increment in the amount of L1 dsRNA was observed, with values of L1/L2 ratio at least four times higher that in the wildtype strains; whereas the amount of L2 and the ratio L2/S1 dsRNAs are similar in S2-cured clones and in wildtype strain. Therefore, the loss of S2 dsRNA in strain UCD 67-385 affect the copy number of L1 but not of L2 dsRNA.

## Discussion

The existence of a high degree of dsRNA viral polymorphism among strains of *X. dendrorhous *isolated from six different regions was determined. In general the results obtained here are in accord with previous reports for isolates from Japan [19,21], but with some differences. We found that strain CBS 5908 have four dsRNA elements, but previous work with the same strain but acquired from different culture collections (see Table 1 for name equivalences) reported no dsRNA (strain UCD 67-383, [21]) or three dsRNAs (strain ATCC 24203, [19]. To test if this variation may have been generated by the subculturing/processing steps of each culture bank, we purchased this strain from the three culture collections (ATCC, CBS and UCD), and determined their dsRNA content. No differences in their dsRNA pattern was observed (L1, L2, S1 and S2 dsRNAs), excluding these as a possibility. The molecular sizes of dsRNA elements reported here are larger than previously reported [19,21], probably due to differences in the estimation method. In this work, each dsRNA molecule was isolated from gels, following were subjected to electrophoresis in 1 and 2% agarose gels, and calculations of size were made in relation to the 1 kb DNA marker correcting the values obtained (average of at least three determinations) according to the method of Livshits *et al*. [24].

Analyzing the dsRNA composition of *X. dendrorhous *strains revealed characteristic patterns related to the locality/region where they were isolated from: Finland, no dsRNA; Moscow region, one dsRNA (L1); Alaska, one dsRNA (L2); Hiroshima, two dsRNAs (L1 and S1); Patagonia, two dsRNAs (L1 and M); and Kiso, four dsRNAs (L1, L2, S1, and S2). Additionally, a genomic variation between L1 dsRNA elements of *X. dendrorhous *strains of different geographic origin was observed, even from near regions (Kiso and Hiroshima). However, the L2 dsRNA of Alaskan strain (UCD 68-653C) hybridized with L1 dsRNAs of Japanese and Russian strains, even though no hybridization between these last two dsRNAs was observed. These results suggest that the nucleotide sequence of Alaskan L2 dsRNA is a mosaic among the L1 dsRNAs from Japanese and Russian *X. dendrorhous *strains. In previous work with other yeasts of the Cystofilobasidiales clade, homology was found between the 5.0 kb dsRNA of *Cystofilobasidium *sp. CBS 6569 and *C. bisporidii *VKM 2700 with dsRNAs from *X. dendrorhous *(strains CBS 5908 and ATCC 24203) [25]. However, according to CBS databases the strain number CBS 6569 correspond to *Dioszegia hungarica*.

The L1 and L2 dsRNA elements of *X. dendrorhous *showed cross-hybridization and they are able to stable replicate as unique elements (Russian and Alaskan strains) and to coexist in the same cell (Japanese strains). Analysis of the relative amount of the four dsRNA elements along the growth curve of *X. dendrorhous *UCD 67-385, revealed an increment of amount of L2 compared with L1, while the amounts of S1 and S2 dsRNAs remained around a fifth of the L1. Furthermore, curing of S2 dsRNA from strain that harbor four dsRNA elements resulted in an increase of amount of L1dsRNA compared with the uncured parental strain, while the amount of L2 and S1dsRNAs was not altered. These results resembled the relationship between the helper virus L and the satellite virus M of the killer system of *S. cerevisiae*, in which an increase of the amount of L dsRNA was observed after elimination of the M dsRNA [22]. Therefore in *X. dendrorhous *the S2 dsRNA would be a satellite virus of L1 dsRNA (helper virus), and probably the S1 dsRNA a satellite virus of L2 dsRNA. Furthermore, the elimination of S2 dsRNA in *X. dendrorhous *was achieved after treatment with anisomycin, a peptidyl-transferase inhibitor that alter the ribosomal frameshifting efficiencies, promoting the loss of yeast dsRNA virus which uses a programmed -1 ribosomal frameshift to produce its Gag-Pol fusion protein [26]. In *S. cerevisiae*, a differential loss of viral genomes has been observed under conditions that cause a decrease in packaging of viral particles, phenomenon described as a preferential cis packaging [27-30]. In this way the viral proteins are preferentially utilized by the helper virus and the satellite virus only can use the excess protein. This preferential packaging mechanism can explain the selective loss of S2 dsRNA and increase in L2 dsRNA in cured strains of *X. dendrorhous*, supporting the hypothesis of the existence of helper/satellite virus system in *X. dendrorhous*.

## Conclusion

The dsRNA elements of *X. dendrorhous *are highly variable in size and sequence, and the dsRNA pattern is almost a fingerprint of the locality/region of isolation of yeast strains. Data presented here suggest that L1 and L2 dsRNA elements of *X. dendrorhous *are homologous viruses able coexist in the same cell, and the existence of at least one helper/satellite virus system in *X. dendrorhous*.

## Methods

### Yeast strains and culture conditions

The yeast strains used in this work are listed in Table 1. Yeasts were grown at 22°C in YM medium (0.3% yeast extract, 0.3% malt extract, and 0.5% peptone) or minimal medium N [31,32] supplemented with 2% glucose or 2% succinate.

### Isolation of total RNA

Yeast pellet (0.1 g) was washed twice with 1 ml of TE buffer (10 mM Tris-HCl, 1 mM EDTA, pH 8.0), resuspended in 0.4 ml of TE, and 0.4 ml of 0.5 mm-diameter glass beads (BioSpec Products, Inc.) and 0.4 ml of acid phenol (equilibrated with 50 mM sodium acetate, pH 4.0) were added. The mixture were vortexed vigorously for 40 min, centrifuged and the aqueous phase was subjected to organic extraction with 1 volume of acid phenol, and twice with 1 volume of chloroform:isoamylic alcohol (24:1). RNAs were precipitated from the aqueous phase by addition of two volumes of isopropanol and incubation at -20°C for 2 h. Samples were centrifuged at 14,000 × *g *and the RNAs were resuspended in 20 μl of nuclease-free water.

### Enzymatic treatments

Samples were treated with DNase I, Nuclease S1 and RNase H, according to standard protocols [33,34]. Digestions with RNAse A were made in SSC buffer (0.3 M NaCl, 0.03 M sodium citrate, pH 7.0) at high (2× SSC) and low (0.01× SSC) ionic strength [21,35].

### Purification and analysis of dsRNA elements

Samples of total RNA were subjected to 1% agarose gels electrophoresis in TAE buffer, stained with ethidium bromide (0.5 μg/ml), and visualized and photographed under UV light. For the calculations of molecular sizes, each of dsRNA elements was purified from the agarose gels using an alternative to glass milk method [36] and resolved in 1 and 2% agarose gels. The values of molecular size were obtained by comparing with λ *Hind*III (Fermentas) and 1 kb (Fermentas) DNA markers using the 1D Image Analysis Software version 2.0.1 (Kodak Scientific Image System), and corrected for the difference in electrophoretic mobility between dsDNA and dsRNA [24].

### Hybridization experiments

Dot blotting: 400 ng of purified dsRNA elements were incubated at 95°C for 10 min in presence of 18% dimethylsulfoxide (DMSO), and immediately cooled on ice. Then were dotted onto a nylon membrane (BiodyneB, Pall) and fixed by incubation at 80°C for 15 min.

Synthesis of cDNA probes: 500 ng of individual dsRNA elements were heat denatured at 95°C for 15 min, and quickly cooled on ice. Then, 200 U of M-MulV reverse transcriptase (New England Biolabs), 1 μl of 25 mM random hexamers, 1 μl of 10 mM nucleotide mix, and 3 μCi of ^32^P-dCTP were added and incubated at 42°C for 90 min. The reaction was stopped by incubation at 70°C for 10 min and cDNAs were purified using Sephadex G-50 columns (GE Healthcare).

Hybridization: membranes were pre-hybridized with 0.5 M NaHPO_4_, 7% SDS, pH 7.2 at 65°C for 10 min, and hybridized with the probe in the same buffer at 65°C for 16 h. Membranes were washed twice with 40 mM NaHPO_4_, 5% SDS, pH 7.2 and twice with 40 mM NaHPO_4_, 1% SDS, pH 7.2, for 30 min at 65°C for each wash. Finally, membranes were exposed to autoradiographic films at -80°C for 4 to 6 days and the films were developed in a Curix 60 system (AGFA).

### Quantification of dsRNAs along the growth curve

Ten ml of a late exponential phase of *X. dendrorhous *culture was added to 500 ml of YM medium and incubated at 22°C with constant agitation. At several times 10 ml of yeast culture samples were harvested. For determination of viable cells, serial dilutions of culture samples were made and aliquots were seeded onto YM agar plate. After incubation at 22°C for 3-5 days, the colony forming units were determined. The total RNA was isolated from each culture sample, subjected to electrophoresis, stained with ethidium bromide and photographed. The intensities of bands corresponding to dsRNA elements were determined using ImageJ software [37]. This semi-quantification was made for various volumes (2 to 10 μl) of each RNA sample, and the comparisons of the intensities of dsRNA elements were made only between samples run on the same gel.

### Curing of dsRNA elements

Aliquots of fresh culture of *X. dendrorhous *UCD 67-385 (DO_600 _~12) was added two fresh medium with different concentrations of anisomycin and incubated at 22°C. The DO_600 _was recorded periodically and 10 ml of the culture were harvested. Aliquots of 100 μl of serial dilutions of culture samples were seeded onto YM solid medium and incubated at 22°C until the development of colonies. Colonies were selected randomly, seeded in 10 ml YM broth and incubated at 22°C for 3 days. The dsRNA elements were determined from each culture as described above.

## Competing interests

The authors declare that they have no competing interests.

## Authors' contributions

MS, participated in the design of this study and carried out the hybridization experiment; VO and OF carry out the RNA isolations and analysis; VC, participated in the study design; DL kindly provided Argentine's *X. dendrorhous *strains and participated in data analysis; MB conceived the study and participated in its design and coordination; and MB and VC wrote the manuscript. All authors approved the final manuscript.
